# 3-(1-Adamant­yl)-1-{[4-(2-meth­oxy­phen­yl)piperazin-1-yl]meth­yl}-4-methyl-1*H*-1,2,4-triazole-5(4*H*)-thione

**DOI:** 10.1107/S1600536810022695

**Published:** 2010-06-23

**Authors:** Abdul-Malek S. Al-Tamimi, Ahmed Bari, Mohamed A. Al-Omar, Khalid A. Alrashood, Ali A. El-Emam

**Affiliations:** a Department of Pharmaceutical Chemistry, College of Pharmacy, King Saud University, Riyadh 11451, Saudi Arabia

## Abstract

The title compound, C_25_H_35_N_5_OS, is a functionalized triazoline-3-thione with substituted piperazine and adamantyl substituents attached at the 2- and 5-positions, respectively, of a triazole spacer with an approximately C-shaped conformation of the mol­ecule. The piperazine ring adopts a chair conformation.

## Related literature

For the anti­viral activity of adamantane derviatives, see: Vernier *et al.* (1969 ▶); Balzarini *et al.* (2007 ▶); El-Emam *et al.* (2004 ▶). For our study of the chemical and pharmacological properties of adamantane derivatives, see: Al-Omar *et al.* (2010 ▶); Al-Abdullah *et al.* (2007 ▶); Al-Deeb *et al.* (2006 ▶); El-Emam *et al.* (2004 ▶). For related structures, see: Hayden *et al.* (1981 ▶); Kadi *et al.* (2007 ▶); Khan *et al.* (2009 ▶); Smith (1969 ▶).
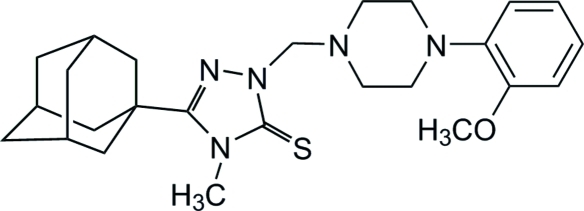


## Experimental

### 

#### Crystal data

C_25_H_35_N_5_OS
*M* *_r_* = 453.64
Monoclinic, 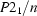

*a* = 11.0203 (2) Å
*b* = 12.1148 (2) Å
*c* = 18.7624 (4) Å
β = 103.473 (1)°
*V* = 2436.01 (8) Å^3^
*Z* = 4
Mo *K*α radiation
μ = 0.16 mm^−1^
*T* = 220 K
0.49 × 0.45 × 0.30 mm

#### Data collection

Bruker APEXII CCD diffractometer
Absorption correction: multi-scan (*SADABS*; Bruker, 2000 ▶) *T*_min_ = 0.657, *T*_max_ = 0.746
24895 measured reflections
5620 independent reflections
3796 reflections with *I* > 2σ(*I*)
*R*_int_ = 0.051

#### Refinement

*R*[*F* ^2^ > 2σ(*F* ^2^)] = 0.044
*wR*(*F* ^2^) = 0.110
*S* = 1.03
5620 reflections
291 parameters
H-atom parameters constrained
Δρ_max_ = 0.24 e Å^−3^
Δρ_min_ = −0.28 e Å^−3^

### 

Data collection: *APEX2* (Bruker, 2004 ▶); cell refinement: *SAINT-Plus* (Bruker, 2004 ▶); data reduction: *SAINT-Plus*; program(s) used to solve structure: *SHELXS97* (Sheldrick, 2008 ▶); program(s) used to refine structure: *SHELXL97* (Sheldrick, 2008 ▶); molecular graphics: *SHELXTL* (Sheldrick, 2008 ▶); software used to prepare material for publication: *SHELXTL*.

## Supplementary Material

Crystal structure: contains datablocks global, I. DOI: 10.1107/S1600536810022695/kp2263sup1.cif

Structure factors: contains datablocks I. DOI: 10.1107/S1600536810022695/kp2263Isup2.hkl

Additional supplementary materials:  crystallographic information; 3D view; checkCIF report

## Figures and Tables

**Fig. 1.**
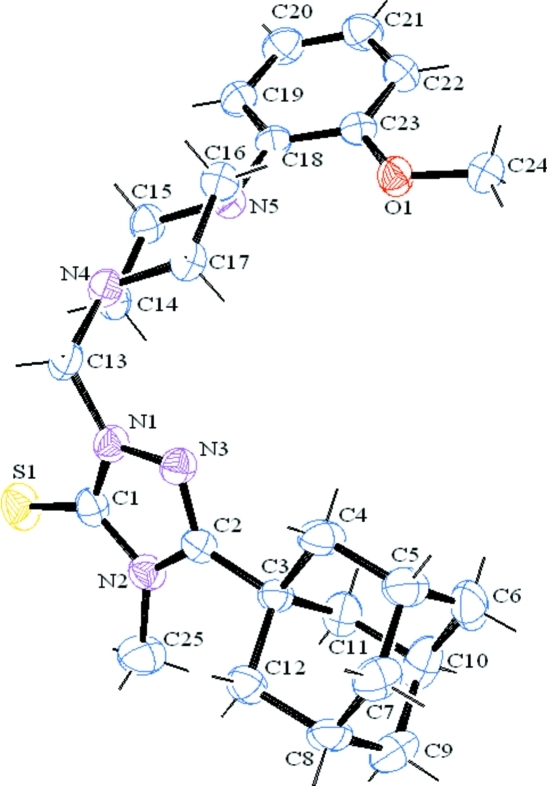
The molecular structure of the title compound, showing 50% probability displacement ellipsoids for non-H atoms and the atom-numbering scheme.

## supplementary crystallographic information

### Comment

Derivatives of adamantane have long been known for their antiviral activity against the influenza (Vernier *et al.*, 1969) and HIV viruses (Balzarini *et al.*, 2007; El-Emam *et al.*, 2004). In continuation to our interest in the chemical and pharmacological properties of adamantane derivatives (Al-Omar *et al.*, 2010; Al-Abdullah *et al.*, 2007; Al-Deeb *et al.*, 2006; El-Emam *et al.*, 2004), we synthesized the title compound as potential chemotherapeutic agent.

### Experimental

A mixture of the 5-(1-adamantyl)-4-methyl-3-mercapto-1,2,4-triazole (0.25 g, 1.0 mmol), 1-(2-methoxyphenyl)-piperazine (0.192 g, 1.0 mmol), and 37% formaldehyde solution (1 ml), in ethanol (8 ml), was heated under reflux for 15 and the mixture was stirred for 12 h at room temperature. The precipitated crude product was filtered, washed with water, dried, and crystallized from ethanol to yield the title compound as colourless crystals.

### Refinement

All H atoms were positioned geometrically and refined using a riding model, with C–H = 0.93–0.99 Å, *U*_iso_(H) = *U*_eq_(C) where *x* = 1.2 or 1.5.

### Crystal data

**Table:**

C_25_H_35_N_5_OS	*D*_x_ = 1.237 Mg m^−^^3^
*M**_r_* = 453.64	Melting point: 368 K
Monoclinic, *P*2_1_/*n*	Mo *K*α radiation, λ = 0.71073 Å
*a* = 11.0203 (2) Å	Cell parameters from 3554 reflections
*b* = 12.1148 (2) Å	θ = 2.2–23.1°
*c* = 18.7624 (4) Å	µ = 0.16 mm^−^^1^
β = 103.473 (1)°	*T* = 220 K
*V* = 2436.01 (8) Å^3^	Irregular, colourless
*Z* = 4	0.49 × 0.45 × 0.30 mm
*F*(000) = 976	

### Data collection

**Table:**

Bruker APEXII CCD diffractometer	5620 independent reflections
Radiation source: fine-focus sealed tube	3796 reflections with *I* > 2σ(*I*)
graphite	*R*_int_ = 0.051
φ and ω scans	θ_max_ = 27.8°, θ_min_ = 2.0°
Absorption correction: multi-scan (*SADABS*; Bruker, 2000)	*h* = −14→12
*T*_min_ = 0.657, *T*_max_ = 0.746	*k* = −15→15
24895 measured reflections	*l* = −23→24

### Refinement

**Table:**

Refinement on *F*^2^	Primary atom site location: structure-invariant direct methods
Least-squares matrix: full	Secondary atom site location: difference Fourier map
*R*[*F*^2^ > 2σ(*F*^2^)] = 0.044	Hydrogen site location: inferred from neighbouring sites
*wR*(*F*^2^) = 0.110	H-atom parameters constrained
*S* = 1.03	*w* = 1/[σ^2^(*F*_o_^2^) + (0.0401*P*)^2^ + 0.5413*P*] where *P* = (*F*_o_^2^ + 2*F*_c_^2^)/3
5620 reflections	(Δ/σ)_max_ = 0.005
291 parameters	Δρ_max_ = 0.24 e Å^−^^3^
0 restraints	Δρ_min_ = −0.28 e Å^−^^3^

### Special details

**Table:**

Geometry. All e.s.d.'s (except the e.s.d. in the dihedral angle between two l.s. planes) are estimated using the full covariance matrix. The cell e.s.d.'s are taken into account individually in the estimation of e.s.d.'s in distances, angles and torsion angles; correlations between e.s.d.'s in cell parameters are only used when they are defined by crystal symmetry. An approximate (isotropic) treatment of cell e.s.d.'s is used for estimating e.s.d.'s involving l.s. planes.
Refinement. Refinement of *F*^2^ against ALL reflections. The weighted *R*-factor *wR* and goodness of fit *S* are based on *F*^2^, conventional *R*-factors *R* are based on *F*, with *F* set to zero for negative *F*^2^. The threshold expression of *F*^2^ > σ(*F*^2^) is used only for calculating *R*-factors(gt) *etc*. and is not relevant to the choice of reflections for refinement. *R*-factors based on *F*^2^ are statistically about twice as large as those based on *F*, and *R*- factors based on ALL data will be even larger.

### Fractional atomic coordinates and isotropic or equivalent isotropic displacement parameters (Å^2^)

**Table:**

	*x*	*y*	*z*	*U*_iso_*/*U*_eq_	
S1	0.85940 (5)	0.86853 (4)	0.53498 (3)	0.05000 (16)	
N1	0.62964 (13)	0.78915 (11)	0.47013 (7)	0.0332 (3)	
N2	0.66463 (13)	0.94832 (11)	0.42976 (7)	0.0339 (3)	
N3	0.52327 (12)	0.81656 (11)	0.41781 (7)	0.0324 (3)	
N4	0.66353 (13)	0.58757 (11)	0.48190 (7)	0.0332 (3)	
N5	0.72913 (12)	0.44922 (11)	0.37340 (7)	0.0308 (3)	
O1	0.59370 (11)	0.40994 (10)	0.23430 (6)	0.0389 (3)	
C1	0.71784 (16)	0.86777 (14)	0.47860 (9)	0.0338 (4)	
C2	0.54684 (15)	0.91303 (13)	0.39379 (9)	0.0300 (4)	
C3	0.45836 (15)	0.97132 (13)	0.33195 (9)	0.0295 (4)	
C4	0.34127 (17)	0.89905 (14)	0.30667 (11)	0.0440 (5)	
H4A	0.3653	0.8264	0.2915	0.053*	
H4B	0.3007	0.8884	0.3474	0.053*	
C5	0.24998 (18)	0.95439 (15)	0.24238 (11)	0.0478 (5)	
H5	0.1755	0.9069	0.2263	0.057*	
C6	0.3130 (2)	0.96989 (18)	0.17898 (11)	0.0579 (6)	
H6A	0.2547	1.0041	0.1374	0.069*	
H6B	0.3383	0.8981	0.1632	0.069*	
C7	0.21012 (17)	1.06595 (15)	0.26620 (11)	0.0445 (5)	
H7A	0.1518	1.1014	0.2251	0.053*	
H7B	0.1677	1.0562	0.3062	0.053*	
C8	0.32449 (17)	1.13792 (14)	0.29152 (10)	0.0393 (4)	
H8	0.2987	1.2104	0.3073	0.047*	
C9	0.38859 (18)	1.15499 (16)	0.22891 (11)	0.0492 (5)	
H9A	0.4624	1.2017	0.2452	0.059*	
H9B	0.3316	1.1921	0.1880	0.059*	
C10	0.42699 (19)	1.04324 (18)	0.20391 (10)	0.0510 (5)	
H10	0.4674	1.0544	0.1625	0.061*	
C11	0.51956 (17)	0.98754 (16)	0.26713 (9)	0.0410 (4)	
H11A	0.5943	1.0335	0.2823	0.049*	
H11B	0.5447	0.9159	0.2511	0.049*	
C12	0.41605 (16)	1.08353 (14)	0.35596 (9)	0.0369 (4)	
H12A	0.3756	1.0730	0.3968	0.044*	
H12B	0.4887	1.1315	0.3728	0.044*	
C13	0.63210 (17)	0.68799 (14)	0.51391 (9)	0.0364 (4)	
H13A	0.6922	0.6988	0.5609	0.044*	
H13B	0.5497	0.6786	0.5244	0.044*	
C14	0.78816 (15)	0.59036 (14)	0.46694 (9)	0.0350 (4)	
H14A	0.8497	0.6098	0.5119	0.042*	
H14B	0.7906	0.6473	0.4302	0.042*	
C15	0.82185 (16)	0.48005 (14)	0.43932 (9)	0.0355 (4)	
H15A	0.9044	0.4845	0.4283	0.043*	
H15B	0.8251	0.4237	0.4772	0.043*	
C16	0.60608 (15)	0.44145 (14)	0.39088 (9)	0.0349 (4)	
H16A	0.6086	0.3857	0.4290	0.042*	
H16B	0.5436	0.4186	0.3472	0.042*	
C17	0.56982 (15)	0.55202 (14)	0.41724 (9)	0.0345 (4)	
H17A	0.5631	0.6070	0.3782	0.041*	
H17B	0.4883	0.5460	0.4295	0.041*	
C18	0.76033 (15)	0.35869 (13)	0.33294 (9)	0.0299 (4)	
C19	0.86273 (16)	0.29100 (14)	0.35965 (10)	0.0367 (4)	
H19	0.9121	0.3034	0.4070	0.044*	
C20	0.89376 (18)	0.20552 (15)	0.31792 (11)	0.0431 (4)	
H20	0.9647	0.1621	0.3367	0.052*	
C21	0.82146 (18)	0.18439 (15)	0.24975 (10)	0.0438 (5)	
H21	0.8403	0.1244	0.2224	0.053*	
C22	0.72050 (17)	0.25144 (14)	0.22101 (10)	0.0387 (4)	
H22	0.6718	0.2377	0.1736	0.046*	
C23	0.69049 (15)	0.33847 (13)	0.26127 (9)	0.0315 (4)	
C24	0.53309 (18)	0.39994 (16)	0.15850 (9)	0.0433 (5)	
H24A	0.5949	0.4026	0.1292	0.065*	
H24B	0.4744	0.4602	0.1445	0.065*	
H24C	0.4888	0.3302	0.1503	0.065*	
C25	0.72686 (18)	1.05429 (15)	0.42605 (12)	0.0517 (5)	
H25A	0.6880	1.1107	0.4500	0.077*	
H25B	0.7194	1.0744	0.3752	0.077*	
H25C	0.8144	1.0480	0.4506	0.077*	

### Atomic displacement parameters (Å^2^)

**Table:**

	*U*^11^	*U*^22^	*U*^33^	*U*^12^	*U*^13^	*U*^23^
S1	0.0391 (3)	0.0588 (3)	0.0435 (3)	0.0019 (2)	−0.0079 (2)	−0.0074 (2)
N1	0.0324 (8)	0.0346 (8)	0.0308 (7)	0.0048 (6)	0.0038 (6)	0.0019 (6)
N2	0.0307 (8)	0.0320 (7)	0.0367 (8)	−0.0009 (6)	0.0034 (6)	−0.0031 (6)
N3	0.0290 (8)	0.0325 (8)	0.0341 (8)	0.0021 (6)	0.0042 (6)	0.0016 (6)
N4	0.0351 (8)	0.0360 (8)	0.0285 (7)	0.0052 (6)	0.0077 (6)	0.0022 (6)
N5	0.0266 (7)	0.0322 (7)	0.0329 (7)	0.0009 (6)	0.0056 (6)	−0.0018 (6)
O1	0.0372 (7)	0.0397 (7)	0.0358 (7)	0.0074 (5)	0.0005 (5)	−0.0026 (5)
C1	0.0338 (9)	0.0376 (9)	0.0297 (9)	0.0024 (8)	0.0068 (7)	−0.0052 (7)
C2	0.0282 (9)	0.0280 (8)	0.0334 (9)	−0.0008 (7)	0.0062 (7)	−0.0036 (7)
C3	0.0285 (9)	0.0252 (8)	0.0332 (9)	0.0012 (7)	0.0042 (7)	−0.0012 (6)
C4	0.0360 (10)	0.0292 (9)	0.0589 (12)	−0.0035 (8)	−0.0049 (9)	0.0027 (8)
C5	0.0369 (10)	0.0348 (10)	0.0601 (13)	−0.0035 (8)	−0.0121 (9)	0.0012 (9)
C6	0.0633 (14)	0.0568 (13)	0.0414 (11)	0.0224 (11)	−0.0125 (10)	−0.0106 (10)
C7	0.0310 (10)	0.0440 (11)	0.0561 (12)	0.0064 (8)	0.0054 (8)	0.0091 (9)
C8	0.0411 (10)	0.0250 (9)	0.0502 (11)	0.0090 (7)	0.0075 (8)	0.0002 (8)
C9	0.0431 (11)	0.0433 (11)	0.0582 (13)	0.0015 (9)	0.0059 (9)	0.0194 (9)
C10	0.0482 (12)	0.0716 (14)	0.0349 (10)	0.0174 (10)	0.0129 (9)	0.0109 (9)
C11	0.0378 (10)	0.0494 (11)	0.0370 (10)	0.0110 (9)	0.0109 (8)	−0.0007 (8)
C12	0.0369 (10)	0.0331 (9)	0.0404 (10)	0.0040 (8)	0.0086 (8)	−0.0062 (7)
C13	0.0432 (10)	0.0400 (10)	0.0273 (8)	0.0064 (8)	0.0108 (7)	0.0046 (7)
C14	0.0313 (9)	0.0407 (10)	0.0309 (9)	0.0010 (8)	0.0028 (7)	−0.0005 (7)
C15	0.0282 (9)	0.0408 (10)	0.0348 (9)	0.0037 (7)	0.0020 (7)	0.0003 (7)
C16	0.0294 (9)	0.0387 (10)	0.0376 (9)	−0.0021 (7)	0.0100 (7)	0.0007 (8)
C17	0.0291 (9)	0.0409 (10)	0.0334 (9)	0.0029 (7)	0.0074 (7)	0.0028 (7)
C18	0.0283 (9)	0.0278 (8)	0.0351 (9)	−0.0012 (7)	0.0103 (7)	0.0024 (7)
C19	0.0369 (10)	0.0350 (9)	0.0371 (9)	0.0036 (8)	0.0066 (8)	0.0032 (7)
C20	0.0418 (11)	0.0361 (10)	0.0510 (11)	0.0136 (8)	0.0098 (9)	0.0046 (8)
C21	0.0515 (12)	0.0342 (10)	0.0483 (11)	0.0071 (9)	0.0172 (9)	−0.0046 (8)
C22	0.0406 (10)	0.0367 (10)	0.0386 (10)	−0.0005 (8)	0.0089 (8)	−0.0054 (8)
C23	0.0277 (9)	0.0296 (9)	0.0377 (9)	0.0000 (7)	0.0088 (7)	0.0019 (7)
C24	0.0473 (11)	0.0431 (11)	0.0362 (10)	0.0027 (9)	0.0032 (8)	0.0049 (8)
C25	0.0412 (11)	0.0404 (11)	0.0662 (14)	−0.0129 (9)	−0.0020 (10)	0.0013 (10)

### Geometric parameters (Å, °)

**Table:**

S1—C1	1.6684 (17)	C9—H9A	0.9800
N1—C1	1.344 (2)	C9—H9B	0.9800
N1—N3	1.3827 (18)	C10—C11	1.529 (2)
N1—C13	1.472 (2)	C10—H10	0.9900
N2—C1	1.373 (2)	C11—H11A	0.9800
N2—C2	1.384 (2)	C11—H11B	0.9800
N2—C25	1.465 (2)	C12—H12A	0.9800
N3—C2	1.300 (2)	C12—H12B	0.9800
N4—C13	1.434 (2)	C13—H13A	0.9800
N4—C14	1.465 (2)	C13—H13B	0.9800
N4—C17	1.463 (2)	C14—C15	1.511 (2)
N5—C18	1.421 (2)	C14—H14A	0.9800
N5—C15	1.458 (2)	C14—H14B	0.9800
N5—C16	1.471 (2)	C15—H15A	0.9800
O1—C23	1.3755 (19)	C15—H15B	0.9800
O1—C24	1.429 (2)	C16—C17	1.514 (2)
C2—C3	1.506 (2)	C16—H16A	0.9800
C3—C11	1.534 (2)	C16—H16B	0.9800
C3—C4	1.540 (2)	C17—H17A	0.9800
C3—C12	1.538 (2)	C17—H17B	0.9800
C4—C5	1.533 (2)	C18—C19	1.390 (2)
C4—H4A	0.9800	C18—C23	1.406 (2)
C4—H4B	0.9800	C19—C20	1.388 (2)
C5—C6	1.523 (3)	C19—H19	0.9400
C5—C7	1.520 (3)	C20—C21	1.364 (3)
C5—H5	0.9900	C20—H20	0.9400
C6—C10	1.520 (3)	C21—C22	1.382 (2)
C6—H6A	0.9800	C21—H21	0.9400
C6—H6B	0.9800	C22—C23	1.381 (2)
C7—C8	1.515 (3)	C22—H22	0.9400
C7—H7A	0.9800	C24—H24A	0.9700
C7—H7B	0.9800	C24—H24B	0.9700
C8—C9	1.519 (3)	C24—H24C	0.9700
C8—C12	1.532 (2)	C25—H25A	0.9700
C8—H8	0.9900	C25—H25B	0.9700
C9—C10	1.525 (3)	C25—H25C	0.9700
			
C1—N1—N3	112.58 (13)	C3—C11—H11A	109.8
C1—N1—C13	127.26 (14)	C10—C11—H11B	109.8
N3—N1—C13	119.89 (14)	C3—C11—H11B	109.8
C1—N2—C2	108.19 (13)	H11A—C11—H11B	108.2
C1—N2—C25	121.52 (14)	C8—C12—C3	109.75 (13)
C2—N2—C25	130.09 (14)	C8—C12—H12A	109.7
C2—N3—N1	104.90 (13)	C3—C12—H12A	109.7
C13—N4—C14	112.91 (14)	C8—C12—H12B	109.7
C13—N4—C17	113.84 (13)	C3—C12—H12B	109.7
C14—N4—C17	111.10 (13)	H12A—C12—H12B	108.2
C18—N5—C15	116.23 (13)	N4—C13—N1	116.57 (13)
C18—N5—C16	114.85 (13)	N4—C13—H13A	108.2
C15—N5—C16	109.13 (13)	N1—C13—H13A	108.2
C23—O1—C24	117.10 (13)	N4—C13—H13B	108.2
N1—C1—N2	103.90 (14)	N1—C13—H13B	108.2
N1—C1—S1	128.52 (13)	H13A—C13—H13B	107.3
N2—C1—S1	127.59 (13)	N4—C14—C15	111.20 (14)
N3—C2—N2	110.43 (14)	N4—C14—H14A	109.4
N3—C2—C3	122.86 (14)	C15—C14—H14A	109.4
N2—C2—C3	126.59 (14)	N4—C14—H14B	109.4
C2—C3—C11	110.21 (13)	C15—C14—H14B	109.4
C2—C3—C4	108.44 (13)	H14A—C14—H14B	108.0
C11—C3—C4	108.67 (15)	N5—C15—C14	109.76 (13)
C2—C3—C12	112.01 (13)	N5—C15—H15A	109.7
C11—C3—C12	109.77 (14)	C14—C15—H15A	109.7
C4—C3—C12	107.65 (14)	N5—C15—H15B	109.7
C5—C4—C3	110.05 (14)	C14—C15—H15B	109.7
C5—C4—H4A	109.7	H15A—C15—H15B	108.2
C3—C4—H4A	109.7	N5—C16—C17	110.09 (13)
C5—C4—H4B	109.7	N5—C16—H16A	109.6
C3—C4—H4B	109.7	C17—C16—H16A	109.6
H4A—C4—H4B	108.2	N5—C16—H16B	109.6
C6—C5—C7	109.61 (16)	C17—C16—H16B	109.6
C6—C5—C4	109.47 (17)	H16A—C16—H16B	108.2
C7—C5—C4	109.82 (16)	N4—C17—C16	110.03 (13)
C6—C5—H5	109.3	N4—C17—H17A	109.7
C7—C5—H5	109.3	C16—C17—H17A	109.7
C4—C5—H5	109.3	N4—C17—H17B	109.7
C5—C6—C10	109.05 (16)	C16—C17—H17B	109.7
C5—C6—H6A	109.9	H17A—C17—H17B	108.2
C10—C6—H6A	109.9	C19—C18—C23	117.34 (15)
C5—C6—H6B	109.9	C19—C18—N5	122.67 (15)
C10—C6—H6B	109.9	C23—C18—N5	119.90 (14)
H6A—C6—H6B	108.3	C20—C19—C18	121.50 (16)
C8—C7—C5	109.12 (15)	C20—C19—H19	119.2
C8—C7—H7A	109.9	C18—C19—H19	119.2
C5—C7—H7A	109.9	C21—C20—C19	120.12 (17)
C8—C7—H7B	109.9	C21—C20—H20	119.9
C5—C7—H7B	109.9	C19—C20—H20	119.9
H7A—C7—H7B	108.3	C20—C21—C22	119.80 (17)
C7—C8—C9	109.79 (16)	C20—C21—H21	120.1
C7—C8—C12	110.30 (15)	C22—C21—H21	120.1
C9—C8—C12	109.24 (15)	C23—C22—C21	120.59 (17)
C7—C8—H8	109.2	C23—C22—H22	119.7
C9—C8—H8	109.2	C21—C22—H22	119.7
C12—C8—H8	109.2	O1—C23—C22	122.90 (15)
C8—C9—C10	109.23 (15)	O1—C23—C18	116.54 (14)
C8—C9—H9A	109.8	C22—C23—C18	120.55 (15)
C10—C9—H9A	109.8	O1—C24—H24A	109.5
C8—C9—H9B	109.8	O1—C24—H24B	109.5
C10—C9—H9B	109.8	H24A—C24—H24B	109.5
H9A—C9—H9B	108.3	O1—C24—H24C	109.5
C6—C10—C9	110.18 (16)	H24A—C24—H24C	109.5
C6—C10—C11	109.55 (17)	H24B—C24—H24C	109.5
C9—C10—C11	109.63 (16)	N2—C25—H25A	109.5
C6—C10—H10	109.2	N2—C25—H25B	109.5
C9—C10—H10	109.2	H25A—C25—H25B	109.5
C11—C10—H10	109.2	N2—C25—H25C	109.5
C10—C11—C3	109.53 (14)	H25A—C25—H25C	109.5
C10—C11—H11A	109.8	H25B—C25—H25C	109.5
			
C1—N1—N3—C2	0.20 (18)	C9—C10—C11—C3	59.7 (2)
C13—N1—N3—C2	174.73 (14)	C2—C3—C11—C10	178.08 (15)
N3—N1—C1—N2	0.23 (17)	C4—C3—C11—C10	59.39 (19)
C13—N1—C1—N2	−173.81 (14)	C12—C3—C11—C10	−58.10 (19)
N3—N1—C1—S1	−179.72 (12)	C7—C8—C12—C3	60.79 (19)
C13—N1—C1—S1	6.2 (3)	C9—C8—C12—C3	−59.97 (19)
C2—N2—C1—N1	−0.56 (17)	C2—C3—C12—C8	−178.88 (14)
C25—N2—C1—N1	174.65 (16)	C11—C3—C12—C8	58.36 (18)
C2—N2—C1—S1	179.39 (13)	C4—C3—C12—C8	−59.76 (18)
C25—N2—C1—S1	−5.4 (2)	C14—N4—C13—N1	60.62 (19)
N1—N3—C2—N2	−0.56 (17)	C17—N4—C13—N1	−67.27 (19)
N1—N3—C2—C3	175.85 (14)	C1—N1—C13—N4	−101.45 (19)
C1—N2—C2—N3	0.73 (19)	N3—N1—C13—N4	84.90 (19)
C25—N2—C2—N3	−173.93 (18)	C13—N4—C14—C15	175.19 (13)
C1—N2—C2—C3	−175.51 (15)	C17—N4—C14—C15	−55.49 (17)
C25—N2—C2—C3	9.8 (3)	C18—N5—C15—C14	168.11 (13)
N3—C2—C3—C11	−117.37 (17)	C16—N5—C15—C14	−60.06 (17)
N2—C2—C3—C11	58.4 (2)	N4—C14—C15—N5	57.71 (18)
N3—C2—C3—C4	1.5 (2)	C18—N5—C16—C17	−166.47 (13)
N2—C2—C3—C4	177.26 (16)	C15—N5—C16—C17	60.98 (17)
N3—C2—C3—C12	120.12 (17)	C13—N4—C17—C16	−175.66 (13)
N2—C2—C3—C12	−64.1 (2)	C14—N4—C17—C16	55.52 (17)
C2—C3—C4—C5	−178.66 (15)	N5—C16—C17—N4	−58.50 (17)
C11—C3—C4—C5	−58.86 (19)	C15—N5—C18—C19	10.3 (2)
C12—C3—C4—C5	60.0 (2)	C16—N5—C18—C19	−118.81 (17)
C3—C4—C5—C6	59.8 (2)	C15—N5—C18—C23	−166.10 (15)
C3—C4—C5—C7	−60.6 (2)	C16—N5—C18—C23	64.78 (19)
C7—C5—C6—C10	60.0 (2)	C23—C18—C19—C20	−1.2 (2)
C4—C5—C6—C10	−60.5 (2)	N5—C18—C19—C20	−177.64 (16)
C6—C5—C7—C8	−60.8 (2)	C18—C19—C20—C21	−1.7 (3)
C4—C5—C7—C8	59.5 (2)	C19—C20—C21—C22	2.9 (3)
C5—C7—C8—C9	60.68 (19)	C20—C21—C22—C23	−1.3 (3)
C5—C7—C8—C12	−59.7 (2)	C24—O1—C23—C22	−7.7 (2)
C7—C8—C9—C10	−59.67 (19)	C24—O1—C23—C18	171.55 (15)
C12—C8—C9—C10	61.40 (19)	C21—C22—C23—O1	177.68 (16)
C5—C6—C10—C9	−59.3 (2)	C21—C22—C23—C18	−1.6 (3)
C5—C6—C10—C11	61.4 (2)	C19—C18—C23—O1	−176.55 (14)
C8—C9—C10—C6	59.1 (2)	N5—C18—C23—O1	0.0 (2)
C8—C9—C10—C11	−61.5 (2)	C19—C18—C23—C22	2.8 (2)
C6—C10—C11—C3	−61.3 (2)	N5—C18—C23—C22	179.35 (15)
